# “Thrown in the deep end”: a qualitative study of barriers secondary school staff encounter when addressing self-harm

**DOI:** 10.1186/s12889-025-22826-w

**Published:** 2025-05-19

**Authors:** Hay Wing Charlotte Chan, Tamsin Ford, Astrid Janssens, Joanna Anderson, Jeff Gavin, Abigail E. Russell

**Affiliations:** 1 University of Bath, Bath, UK; 2 Department of Psychiatry, University of Cambridge, Cambridge, UK; 3 Julius Center for Health Sciences and Primary Care, University Medical Center Utrecht, the Netherlands, Utrecht, Netherlands; 4 Department of Psychiatry, University of Cambridge, Cambridge, UK; 5 Department of Psychology, University of Bath, Bath, UK; 6 Department of Public Health and Sports Sciences, Child and Adolescent Mental Health and NIHR Advanced Fellow, ChYMe, University of Exeter Medical School, South Cloisters, St Luke’s Campus, University of Exeter, Exeter, UK

**Keywords:** Self-harm, Schools, Qualitative, Barriers, Support

## Abstract

**Background:**

Self-harm is highly prevalent among young people yet remains misunderstood and stigmatised in schools and among pupils. Schools are positioned to first detect self-harm but are ill-equipped to respond or support. Despite these concerns, studies exploring the management of self-harm in schools from staff perspectives are limited.

**Methods:**

Therefore, the current study explored experiences of secondary school staff when addressing self-harm in schools through a Thematic Analysis of semi-structured focus groups.

**Results:**

Analysis revealed an overarching theme—addressing self-harm in schools is a systemic issue that requires governmental, institutional, and interpersonal support. Two main themes and five subthemes were identified within this overarching theme.

**Conclusions:**

Lack of standardised guidelines and stigmatisation around self-harm are key barriers that prevent staff from effectively addressing self-harm. Training is crucial for school staff to respond safely to self-harm and avoid fearful or avoidant responses, alongside increased access to clinically trained professionals. These findings are discussed in relation to school-based interventions targeted towards self-harm.

**Supplementary Information:**

The online version contains supplementary material available at 10.1186/s12889-025-22826-w.

## Introduction

Self-harm is the strongest predictor for suicide and the leading cause of serious injury and hospitalization amongst children and young people [1]. One in four young people report self-harm in the UK by 17 years old [2]. Worryingly, self-harm remains stigmatised and misunderstood in schools [3]. Self-harm refers to any act with a non-fatal outcome where an individual engages in a behaviour or ingests a substance with the intention of causing harm to themselves [4]. Schools are crucial settings for detecting and supporting youth mental health difficulties [5]. Early identification of suicidal thoughts or self-harm behaviour is crucial [6]. Self-harm makes up 5.1% of all reported safeguarding incidents in schools [7]. Furthermore, the UK Government has set out expectations for schools to play a key role in improving mental health service delivery for young people [8]. Schools are on the frontline to identify youth self-harm as 36% of students reported that they would go to a teacher if they needed mental health support while 30.4% would speak to a school counsellor [9]. Trusted and supportive authority figures, such as school staff, are critical to encourage adolescents’ help-seeking [5].

A recent meta-analysis showed that evidence-informed guidelines can assist school staff to provide better support and respond to student self-harm [10]. Training of school staff may provide them with the knowledge and confidence to respond appropriately to self-harm and prevent escalation that requires emergency intervention. Yet, this is not always provided. In a recent survey, only half of school staff reported receiving some form of training on adolescent self-harm and only 22% of these rated the training as highly adequate [11]. Self-harm is often rendered invisible [12], and young people who disclose self-harm are persistently stigmatised, often perceived as “attention-seeking” or “manipulative” [13]. Teachers who report limited knowledge of self-harm often react with strong negative emotions like “shock” and being “freaked out” when they detect self-harm in students [14]. Stigma and misunderstanding could foster this cycle of shame and guilt [10]. Stigma of self-harm can lead to social isolation from parents and friends, and symptoms of depression, thus further hindering future disclosure or help-seeking behaviour [15].

Evans et al. [11] explored the current delivery of self-harm intervention in school contexts, and barriers to implementation, finding schools would prefer programs that promote mental health awareness or wellbeing, over targeted interventions on self-harm whilst mental health training was not mandatory for school staff. However, they did not explore school staff’s experience addressing self-harm in school. To the best of our knowledge, no qualitative study has been conducted to understand how UK schools are currently managing and responding to students who self-harm. The current study aimed to (1) identify the perceived barriers school staff encounter when addressing self-harm and (2) make practical recommendations for schools to effectively support students who self-harm.

## Methods

### Design

This study was a secondary analysis of qualitative data from secondary schools in South-West England (*n* = 4) and Wales (*n* = 4) as part of a wider project that examined the existing provision of student self-harm prevention and intervention activities. Evans et al. [11] reported the survey findings: this study reports the previously unpublished analysis of the qualitative data from focus groups.

### Participants and sampling

Eight focus groups were conducted with a total of 47 participants. Each focus group included staff from one school, the number of participants in each group ranged from two to nine. Three of the focus groups comprised five or six staff members. In the fourth focus group, the school encountered some organisational problems and following the rearranging of the focus group only two staff members were able to attend. The professional roles of staff included: assistant head teacher; Special Educational Needs Co-ordinator (SENCo); school counsellor; head of house/year; teacher; teaching assistant; safeguarding officers; and pastoral or support staff. Schools were purposively recruited to represent a breadth of key characteristics: self-harm provision, free school meal eligibility (above or below regional mean) and geographical spread. All focus group participants were staff members from schools that completed the initial quantitative survey and indicated interest in follow-up participation [11]. All focus groups were conducted in school, and schools were reimbursed £200 for their time. Informed consent was obtained from all participants.

### Ethical approval

Ethical approval was granted for this study by the University of Cardiff (SREC/4104) and the University of Bath (UG 21-033).

Clinical trial number: not applicable.

### Procedure

Facilitators followed a semi-structured interview schedule (Supporting Information S1) that covered schools’ current approach toward students who self-harm, future intervention needs and recommendations for new practices. Focus groups lasted one to two hours and were audio-recorded then transcribed verbatim. AR led half of the data collection and was involved with generation of transcripts. The other focus group facilitator was a trained mental health practitioner. Audio recordings were not obtained due to identifiable information however the transcripts used for analysis were checked for accuracy and anonymised.

### Data analysis

Author CC led the data analysis. Weekly meetings were held to discuss the coding process and development of thematic map with AR who collected 50% of the original data and was heavily involved in the wider project, having previously coded all the qualitative data to answer other research questions. Several meetings were arranged to discuss emerging findings with the wider research team to ensure themes and subthemes were perceived as accurate in relation to the data.

Data were analysed using Thematic Analysis [16]. A coding tree was developed from an initial descriptive labelling of transcripts line by line. This was discussed and refined with a researcher from the original team. The transcripts were coded using the coding tree structure in NVivo version 12, with new codes being added, combined, and refined in an iterative manner. A thematic map that captured the initial codes was developed after the full dataset was coded (See Supporting Information S2). Themes were then generated on a semantic level using an inductive approach [16]. After further revisiting the original transcripts, themes were refined and finalised.

### Reflexivity

Author CC acknowledge that they approach the study analysis from the position of an undergraduate psychology student at University of Bath, where mental health knowledge is accessible. Furthermore, AR collected the data and was familiar with the tone and expression of staff participating in focus groups. She and the other authors associated are accomplished researchers in the field of child and adolescent mental health, with high education levels and pre-existing proficiency.

## Results

**Fig. 1:**
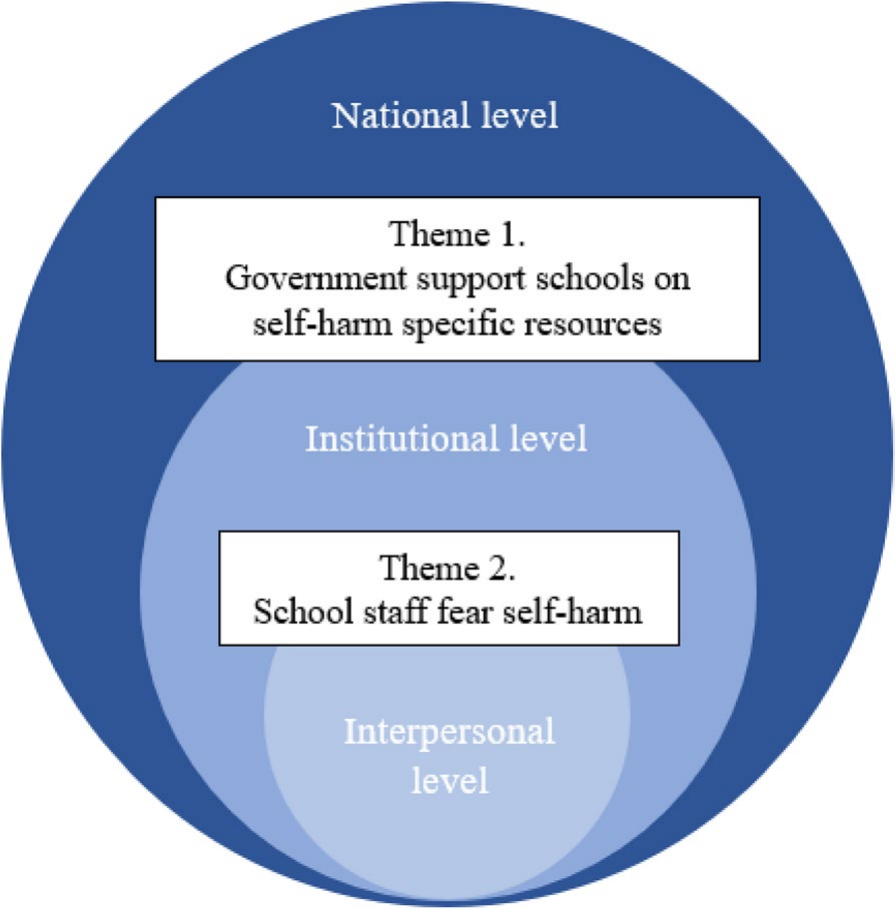
Two main themes in terms of systemic levels they operate across

An overarching theme across the whole dataset was that the management of self-harm in schools is a systemic problem. Schools need resources and guidance that are specific to self-harm, as currently staff lack knowledge and confidence. This fosters fear of addressing and managing self-harm in schools. The subthemes all play important and interacting roles in the same ecological system instead of being discrete factors that stand alone. Within the overarching theme, two main themes were generated (see Table 1). Theme one represents barriers to self-harm management that interact at the national level (government) and the institutional level (schools). Theme two represents barriers that interact across with the institutional level (schools) and interpersonal level (individual staff members). Figure 1 demonstrates how the two themes align in an ecological system.

**Table 1:** Themes and subthemes identified in relation to the study research questions

Themes	Subthemes
Government support schools on self-harm specific resources	• Standardised safeguarding procedures for self-harm • Access to mental health professionals • Access to professional training on safeguarding knowledge
School staff fear self-harm	• Fear of contagion • Stress and demands on staff o Emotive nature of self-harm o Conflict in roles and responsibility

## Theme 1: Government support schools on self-harm specific resources

Self-harm was perceived by participants as the responsibility of schools, falling under the *“safeguarding umbrella”.*

### Standardised safeguarding procedure for self-harm

All staff reported that there was no standardised safeguarding procedure provided by local authorities or the UK government *“…If we were talking about a self-harmer we didn’t have a strategy… “.* Staff reported that a standardised guideline would be helpful:

*“Everybody should follow the same… same paperwork, same everything…”*

Schools generally followed their safeguarding procedure. but stated that they would prefer a guideline specific to self-harm since they do not consider themselves *“mental health experts”* and felt they “*don’t have the expertise within to be able to talk about self-harm”*. Multiple participants said they relied on third sector organisations to look for strategies but were unsure of how appropriate resources were: *“We want to cover ourselves in terms of safeguarding…but I don’t know if what I source off the internet is the correct document.”* Many discussed creating a *“chain of support”*. Schools varied in designation of who should take responsibility for the student (designated safeguarding leads vs those who had an existing relationship with the student). Instead of one member of staff handling the situation, participants also reported passing the case on a small group.

### Access to mental health professionals

Participants often lacked information about students’ safety outside of school due to limited communication with emergency services, leading to a delay in staff being aware of additional support needs:

*“Sometimes we don’t even know about it, they just turn up and … they’ll say, “I’ve been in hospital over the weekend Miss, I tried to take my own life.”*

Specifically, participants expressed dissatisfaction with Child and Adolescent Mental Health Services (CAMHS) being* “stretched”.* Schools reported two levels of difficulties with CAMHS: long waiting lists, and difficulties accessing to professional safeguarding advice if referral was not possible. Staff expressed concern over the lack of capacity in CAMHS.

A further consideration was that students might not reach any mental health services since they *“do not meet the threshold”* of a *“high-end case”,* even after onset of self-harm or suicide attempts. Participants reported that students often do not get *“picked up because they’ve not taken it to the extremes”.* Participants reported struggling to both access and communicate with CAMHS. They also sought help from other professionals in schools, such as “*counsellors”* and *“educational psychologists”*. However, most referred to these professionals as infrequently present, or unavailable, only having support for their school for e.g. “*6 h a week”*.

### Access to professional training on safeguarding knowledge

Many reported a lack of confidence addressing self-harm. School staff being equipped with knowledge was perceived to be key to being able to provide better support:

*“We sort of need a knowledge and information sharing session, but not the fear.”*

Concerns derived from inexperience and fear that inappropriate language or vocabulary would worsen the situation. This was exemplified in their discussion of perception of self-harm. Some participants reported that they were concerned with the reasons behind self-harm, attributing self-harm as a *“negative coping mechanism”,* or “*self-destruction”*.

Staff felt they lacked access to professional advice on self-harm, and a *“mental health care plan”* tailored for school staff on “*what were the good things and the things not to do”* would be useful. Training or workshops were reported to be desirable, yet few reported receiving any training:

*“I’ve not had any training how to deal with that other than you know we do a risk assessment?”*

For staff who had received training, programmes were often not designed for secondary schools, self-harm specifically, or were delivered infrequently.

## Theme 2. School staff fear self-harm

### Fear of contagion

Many participants reported that their school avoided addressing self-harm directly with students. Self-harm was considered a *“taboo subject”* that was not discussed openly. Staff feared that discussion would *“put the idea in their head*”, providing students with information that would not be accessible otherwise. Any open discussion about self-harm was thought to potentially encourage it: *“I feel really uneasy about that because most of the kids in year 11 aren’t doing that”.* Staff instead considered wellbeing workshops or mental health-promoting activities as appropriate interventions that could prevent self-harm. Assemblies or events on “*mindfulness and counselling”* were delivered as a part of the curriculum and are universal programs delivered to foster “*emotional coping mechanisms”* rather than specifically addressing self-harm or suicide.

Few participants had different perspectives on addressing self-harm amongst students. Most showed understanding that disregarding self-harm in schools was an ineffective way to prevent it. They suggested that self-harm could be addressed openly if it were done in a sensitive manner. One participant suggested that, if self-harm can be explained to students by professionals, students could then learn *“how to manage how [they] feel.”*

One group was an exception to the other schools in the study: staff reported that encouraging discussion of self-harm has *“worked wonders”* for them as they shifted the school policy to raise awareness of mental health concerns and encourage help-seeking behaviour.

*“It’s still a bit of a taboo subject … but it’s ok for them to be able to come to us and say, ‘I’ve had this urge to do this’ or ‘I have done this’ instead of there still being this whole conception about it being a cry for attention.”*

The group reported that there were “*less incidents of self-harm” and “less copy-cat things”* after the change in approach and introduction of transparency on the subject:

*“In its isolation is that the reason why it’s gone down? Probably not. But is it a contributing factor? Yes I think it is.”*

However, this differed starkly with other focus groups, where participants were concerned that any group-level discussion of self-harm could be harmful, especially as it might not stay confidential among peers.

*“Kids are kids…it wouldn’t stay confidential…some of their private things would be then divulged down the corridor.”*

Students learning about self-harm from peers was another concern reported by participants in relation to their reluctance. Staff expressed concern over vulnerable students who they felt may start to self-harm if this was a norm in online friendship groups: “*They are looking to belong to a group, to conform.”* Participants reported that peers often were the first to detect self-harm among others and were concerned that this detection might impact on other students’ wellbeing. Some expressed their concern as to whether self-harm was too *“raw”* and *“heavy”* to be discussed among students: *“I think it’s the worry of the individual but also the impact that it has on others around that individual.”* Participants were also concerned that detection and unsupervised discussion between peers on self-harm would potentially introduce additional and unnecessary stress on students.

*“…say she is about to self-harm, call up one of their friends…absolutely scare the living daylights out of the friend … “*

Overall, staff reported that they preferred to work with other staff to gain more safeguarding knowledge and support individual student disclosures. Participants perceived adults *“react better”* to self-harm information.

### Stress and demands on staff

#### Emotive nature of self-harm

Participants expressed that they had experienced negative emotional states like *“fear”* or *“panic”* when they had to manage self-harm. Self-harm was considered an emotionally challenging topic and their limited knowledge only amplified those emotions:

*“I don’t want to take my eye off the ball and then think, ‘Oh if I’d only done this, she may have not done that’.”*

Participants reported that handling self-harm had a negative impact on their personal lives and found it “*exhausting”*:*“For my sanity I have to separate my job from my home life because I’d go mad.”* Most reported their school instructed staff not to panic when self-harm incidents arose, but some reported to have failed to do so:

*“…both teachers one after the other came dashing down … and were really quite frightened by it, panicked…because they don’t want to say the wrong thing.”*

Participants expressed that the rapport they build with the students could become a challenge, as these in-depth personal discussions lead to difficulties maintaining a “*teacher-student boundary”*, reporting having difficulties setting clear boundaries. A few mentioned that they experienced this blurring of role especially when they accompanied the student to medical settings: *“…she wouldn’t go with her mother without [staff] there…”.* Staff also expressed their concerns about losing students’ trust when they followed school policy and informed other teachers or parents.

*“We have a duty of care to tell the parents so we’re stuck between a rock and a hard place.”*

Communication with parents was perceived by staff as one of the most difficult aspects. Parents could be “*defensive”* or *“aggressive”* when they were informed;

*“I’d say, sometimes…the parents aren’t particularly understanding..And…it makes the students more anxious…and actually that that can trigger more self-harm”*

#### Conflict in roles and responsibility

Some participants, especially pastoral staff, reported that managing self-harm should not be the responsibility of subject teachers, as their remit was academic.

*“I think the problem for staff is they still don’t feel it’s their job to deal with mental health”*

However, this varied, with the majority feeling that involvement in mental health training or workshops should be part of all staffs’ responsibility, and the workload and responsibility for each individual should vary depending on job role and personal preference. As mentioned earlier, procedures within schools to manage self-harm were often to pass on relevant information to one or two key individuals (usually the safeguarding lead).

## Discussion

Our findings complement and extend existing research that indicates youth self-harm demands immediate response from schools; yet systematic barriers such as a lack of guidance and resources prevent schools from managing self-harm in an appropriate manner [17]. Studies from Canada and Australia have reported similar findings. They showed that it is common for staff to be overwhelmed or find the concept of self-harm in schools “horrifying” [18]. One factor that this qualitative study did not explore was teaching experience, however other research has found that the number of years working in schools was found to be positively associated with better knowledge and confidence to address students’ self-harm [19]. Findings indicate that mental health provision varied across schools. School staff have emphasised the strong need for consistent, regular attendance of mental health professionals to provide immediate support for students [20]. However, despite 81% of school counsellors having reported working with youth self-harm, only 14.8% of psychologists who work in schools report screening students in the previous 12 months (Burn & Rapee, 2021). These concerns do not only stem from school staff: psychologists in the above study expressed concerns over the difficulties supporting students who were identified as being at-risk of self-harm and ability implement support programs. Our findings are consistent with Berger et al. [19] where staff reported difficulties accessing mental health services for students. Counsellors have also identified difficulties in working with students who self-harm due to the lack of training, cooperation with school personnel and school policies [21]. Student self-harm is not within the service boundary of many new practitioners [22], creating an even wider gap between the awareness or disclosure of self-harm in the school setting, and access for that young person to any kind of clinical support.

The current study found that school staff need policies and resources to move forward and to manage self-harm better and more efficiently. The lack of standardised safeguarding procedures for self-harm is a prominent issue reported by staff across different schools [11]. A recent systematic review identified only one high-quality clinical practice guideline for long-term management of self-harm [23]. Implementing national guidelines or policies for schools and other youth settings to follow regarding self-harm or suicide ideation would promote consistency and provide school staff with reassurance. Addressing self-harm in an appropriate and sensitive manner has been shown to help build awareness among young people and support prevention [19]. Findings from this study suggest that awareness of and access to resources such as “*Responding to Issues of self-harm and thoughts of suicide in young people*” published by the Welsh Government [24] would be helpful to staff. This guidance was developed with the intention of providing information for school staff or health professionals on how to respond to self-harm in young people following the data collection for the current study, so it is unknown whether this has been disseminated and implemented within all Welsh schools. Supporting Information S3 details more protocols from different local authorities that educational staff may find of use.

The current study also found that school staff would welcome training on addressing self-harm in young people. A recent systematic review found that interventions aiming to increase skills and confidence of staff to address self-harm are highly effective and acceptable [25]. Staff training is generally viewed as more acceptable and feasible than screening programs for students [26]. Programs like Skills Training on Risk Management (STORM) found improved attitudes and confidence of staff to manage self-harm in schools after a 6-months training [27]. Supporting Information S4 shows more training programs provided by different charity sectors or non-governmental organisations on how to respond safely to youth self-harm in the UK. Given the above evidence showing that training is both effective and acceptable, it is of concern that it is so poorly disseminated to UK school staff. However, additional training poses time, cost and resources concerns that schools currently must bear themselves. Therefore, the government or local education authorities should allocate resources to improve the accessibility of training programs for schools and consider mandating training for those work in safeguarding. Other countries have had success with investing resources in prevention, for example the National Self-Injury Project in Sweden, a collaboration between the Self-harm and Eating Disorders Organisation and the Swedish government have demonstrated success in decreasing the number of young people who self-harm by implementing better and more structured early care such as the Emotion Regulation Individual Therapy for Adolescents [28].

Staff have reservations about delivering universal interventions focusing solely on self-harm as it is perceived to be contagious. Our findings are consistent with the current evidence-base where over 80% of young people would choose to disclose self-harm to their peers (over teachers or parents) [29]. However, studies on social contagion of self-harm in young people produce mixed evidence [30], and as there is little evidence of contagion effects when students were asked about self-harm and suicide [31]. Several empirical studies have found that social modelling or learning about self-harm could increase the likelihood for young people to engage in self-harm [32], yet it should be noted that no causality should be inferred between the onset of and exposure to self-harm. Many factors that could mediate the effect and disclosure may also be nuanced: such as the nature of friendship and their perception of self-harm [33]. There may also be key protective effects when discussing self-harm: having supportive friends and family was associated with a reduced likelihood of engaging in such behavior [34]. Schools’ reluctance to address self-harm poses significant barriers to effective communication, and provision of empirical evidence to allay staff concerns is a high priority for future research.

Our findings also highlighted how young people interact outside of the school environment; social media could be an important source of information for staff. Staff reported that students would frequently encounter self-harm images online, and they are concerned over social contagion online. A content analysis on Instagram identified a high frequency of pictures and posts of self-harm with hashtags like #cutting attached [30]. Online content relating to suicide or self-harm is often described as a “double-edged sword” depending on the way it is portrayed [35]. Specific forms of media reporting of suicide are known to increase risk of imitational suicides, known as the Werther effect [36], but safe reporting also serves as a protective factor, or young people finding supportive peers and safe online space to express their emotions, known as the Papageno effect [37]. Individuals who focussed on the images or methods of self-harm were more likely to engage in self-harm while individuals who sought help were positively influenced [38]. Given that online safety is taught in schools and considered part of their educational remit, extending this to sensitive topics such as self-harm has the potential to benefit young people.

Furthermore, findings from this study indicate that staff were reluctant to talk about self-harm with young people. They hold different views on the subject and many consider open discussion of self-harm to be taboo. Staff clearly indicated their preference to promote preventive programs that do not address self-harm directly, that would have fewer barriers to implementation but may not be as effective in addressing self-harm specifically. Programs such as the Developing Emotional Awareness and Listening (DEAL) from Samaritans, aim to develop emotional awareness and build resilience [39]. Alternative coping skills could be introduced to pupils who self-harm to reduce or avoid self-harm. However, there are few universal prevention programs for self-harm behaviours compared to interventive programs. To the best of our knowledge, there are currently only a few evidence-based school prevention programs such as The Signs of Self-Injury (SOSI) program in the USA [40] and the Du un deine Emotionen (DUDE, [You and your emotions]) program in Germany [41]. Both programs aim to educate staff and students on self-harm while providing guidelines to help respond safely to self-harm. Muehlenkamp et al. [40] found that participation in these programs increased knowledge on self-harm and perceived ability to support peers who self-harm. However, evidence is sparse and no other behaviour such as help-seeking behaviour were measured as outcomes [42]. Further, the SOSI program is a tertiary prevention program that only intervenes after the onset of self-harm. Primary programs, such as the Saving and Empowering Young Lives in Europe (SEYLE), aim to prevent onset of self-harm or suicide yet are rarely evaluated so the effectiveness of those programs remains unclear [43]. Therefore, greater focus should be put on evaluating preventative measures in schools.

The fear of social contagion discussed by our participants might not reflect the reality of adolescents’ experiences, and comparatively little is known from research about how young people discuss about self-harm and suicide. Future research might consider involving young people’s views on school programs to ensure inclusivity and applicability.

### Strengths and limitations

This study bridged the understanding of school staff’s experiences and beliefs on addressing self-harm with their standard practice in school. It also added to the limited qualitative evidence on experiences of staff addressing self-harm, which has not been explored in the UK context. The findings emphasised that resources and training are urgently necessary for schools to respond safely to youth self-harm. However, inherent to the nature of secondary analysis on already existing data, the data were not specifically collected to answer the second research question of this current study [44]. Evans et al. [11] aimed to map the existing provision of student self-harm prevention and intervention activities in schools, while this study focused on the barriers that secondary school’s staff experienced addressing self-harm in schools and practical recommendations. The lead author did not engage in the data collection process and had no control over what was contained in the dataset, they are therefore independent from study-specific nuance or bias [45].

The sample for the study was varied: a wide range of participants were recruited with schools varying in socioeconomic profile and self-harm provision. Participants are with a wide range of different roles within schools, including pastoral staff, subject teachers, teaching assistants, administrative staff and SENCos. This allowed inclusive opinions and views to be captured in focus groups resulting in a broad perspective on how different staff handle self-harm. Although qualitative findings are not necessarily generalisable to other regions of the UK, this study found similarities across Wales and South-West England [46]. Larger-scale research is needed to investigate if the findings are generalisable, and if the same barriers are present in secondary schools across the UK.

The dataset used in the current study was collected in 2016. The UK Government Green Paper on managing youth mental health difficulties has ssince set expectations for schools and clinicians to better support young people’s mental health [47]. However, it is unlikely that major changes were made since recent evidence still showed under-provision of mental health services for young people and an absence of “mental health leads” in schools [8, 48], and as mentioned above for many of these new teams, self-harm is considered too complex for their service remit. Young people with mental health concerns are consistently reporting distressing emotions and a sense of isolation [49].

There is also the potential of selection bias: recruitment of participants was constrained by the existing provision of self-harm prevention and intervention activities reported in the survey that preceded focus group invites, and a school staff gatekeeper supported recruitment of staff to attend the focus groups. It is likely that staff who were more knowledgeable on self-harm management were recruited as participants which may have restricted the variety of perspectives gathered. Participants were recruited on a voluntary basis if they were interested and free at the time arranged by senior staff. This might limit responses from staff who were interested yet not approached or were not able to participate due to the time of the focus group, which was dictated by senior staff.

## Conclusion and recommendations

Based on the current literature and the implications of our finding, we make the following recommendations:

**Table:** 

National level:	1. Allocate mental health expertise in schools that have the knowledge and training to address self-harm (Clinical psychologists, wellbeing practitioners or counsellors) 2. Develop and mandate standardized safeguarding guidelines or protocols on self-harm specifically 3. Develop policy tools specific for self-harm 4. Allocate resources for all schools to be able to train staff on self-harm and suicide
Local community level:	5. Develop safeguarding guidelines or protocols on self-harm that are relevant to the local community 6. Work with researchers or scientists to provide evidence-based intervention or evaluate emerging services for adolescents in need 7. Develop quick and easy-to-access programs that young people and schools could access when youth do not meet the threshold for severity for NHS referral
School level:	8. Encourage staff to participate in training that provides mental health education on self-harm and how to address self-harm in a sensitive manner
Individual staff/student level:	9. Report to school authorities or trusted adults self-harm incidents

Based on the current literature and the implications of our findings, we believe that these recommendations are well-founded. Given the important yet stigmatised nature of self-harm, alongside the complex hierarchical nature of school procedures and governance, with intersecting responsibility of the individual school, Local Authority or Trust, and national policy, resources deployed at all levels are crucial to increase skills and confidence of staff in addressing self-harm [25]. Future research should focus on the feasibility and potential to provide more mental health services for adolescents in school settings that have the remit of addressing self-harm.

In conclusion, this study offers insights into the experience and views of school staff who are supporting young people who self-harm. Staff reported that they feel they lack knowledge and confidence in this area thus hindering appropriate responses to self-harm. Training should be implemented to educate staff on how to safely respond to self-harm, while intervention programs and preventive measures should be developed and evaluated. Increased integration of mental health and clinical specialists in schools who are trained to respond to student self-harm and support school staff is essential. Inclusive policies and guidelines should be developed for local communities and schools to better support young people who self-harm to obtain accessible treatment and address mental health difficulties effectively.

## Key points and relevance

**Table:** 

• Self-harm is the strongest predictor for suicide and highly prevalent amongst children and young people. Yet, self-harm remains stigmatised and misunderstood in schools	
• Schools are positioned to first detect self-harm but are ill-equipped to respond or support	
• This study bridged the understanding of school staff’s experiences and beliefs on addressing self-harm and added to the limited qualitative evidence in the UK context	
• Addressing self-harm in schools is a systemic issue that requires governmental, institutional, and interpersonal support	
• Providing staff with training on addressing self-harm in young people and building a standardised procedure could aid staff to safely respond to self-harm	
• Based on our findings and current evidence, we made practical recommendations for schools to effectively support students who self-harm	

## Supplementary Information

Supplementary Material 1.

## Data Availability

Data that supports the analysis of this study are available upon request. Data are available from University of Cardiff.
