# Supplementary material for: “Thrown in the deep end”: a qualitative study of barriers secondary school staff encounter when addressing self-harm
Source: BMC Public Health. 2025 May 19;25:1836. doi: 10.1186/s12889-025-22826-w (PMC12087240; doi:10.1186/s12889-025-22826-w)
Supplement: Supplementary file 1 — Supplementary Material 1. [file 12889_2025_22826_MOESM1_ESM.docx]

# Supporting Information

## **Supporting Information S1: Interview schedule**

**Draft Topic Guide: Focus Groups and Interviews with School Staff**

Note: Main questions and topics to cover accompanied by prompts if required (italics: things for the facilitator to ensure are discussed through the focus group where appropriate).

Facilitator introduction (rough guide): Name, bit of background/ice breaking etc.

The aim of this focus group is to find out your experiences with pupil self-harm in your school along with how you address it. We plan to develop an intervention for schools and would like to get your thoughts on what this might look like and what would be feasible. We have a range of topics we would like to discuss with you today but feel free to raise other issues that I don’t directly ask about. Does anyone have any questions before I start recording?

1. Initial questions, introductions:
   - Name, role, briefly outline your experience with self-harm in schools
   - In general how would you (as a member of staff/as a school) detect if/when a student is self-harming?
   - *How often are participants confronted with instances of self-harm?*
   - *What is school history with self-harm?*
2. What are you (as a school) currently doing for self-harm awareness and prevention?
   - Prompts from school survey responses
   - *why particular activities/what is good about what they do?*
   - *ensure both prevention and intervention discussed if not spontaneously differentiated*
   - *potential barriers and facilitators: e.g. how well did that work? why did you stop using posters?*
   - Are current provisions sufficient given the needs of your school?
3. What is the governance structure around self-harm in your school?
   - *School policy- is there one*
   - *Council policy*
   - *Taboos/school ethos*
4. Based on your experiences, what role do you think schools have in preventing or intervening with students’ self-harm?
   - Why/why not
   - Whose role is it?
5. Who delivers existing provisions around self-harm? What kind of support do you get around self-harm?
   - Internal support
   - Support for pupils
   - Support for staff dealing with disclosures/incidents
   - External support
   - Linking point: is that sufficient for your/the school’s needs?
6. We are thinking about developing an intervention aiming prevent self-harm in students.
   - From what you already do, what aspects have been positive/ worked well/ had impact on staff/pupils?
   - What did not work? Is there anything you used to do but stopped? *(barriers and facilitators) and if it didn’t work or get off the ground as it wasn’t feasible, why was that?*
   - What should an ideal SH intervention look like? (and what would be feasible within current school context?)
     - *(devil’s advocate e.g. if ethos of keeping it quiet, would they be prepared to change this for a new intervention emphasising reducing stigma/being open about things)*
   - Who might be involved in a successful intervention? E.g. Part of the development of the intervention / delivering an element of the intervention / …
     - Parents
     - Pupils
     - External health professionals
     - Community/local groups
     - Anyone else?
7. *(linked in with 6 as needed / in case we need to provide structure and generate ideas)*

Are there other programmes within the school that work well e.g. for bullying, alcohol, smoking?

- - What other problems have you been able to address successfully?
  - What worked?
  - Who was involved?
  - When thinking about an intervention for self-harm which of these strategies do you think might be successful? (why?)
  - *Any other ideas for a self-harm intervention not within current school provisions?*

1. Does anyone have any further points they would like to add?

**Additional Prompts**

*Other points for facilitator to be aware of and ask about throughout focus group:*

- *Prevention or reaction*
- *Barriers and facilitators*
- *Following up details e.g. if mention leaflets on self-harm, where did they source them from etc, who do the staff get their info from?*
- *Who is responsible for dealing with self-harm e.g. is the school responsible or prepared to be responsible, or do they view as external issue and want to refer incidents to other agencies (why)*
- *Pupil involvement at all levels*

## **Supporting Information S2: Thematic Map**


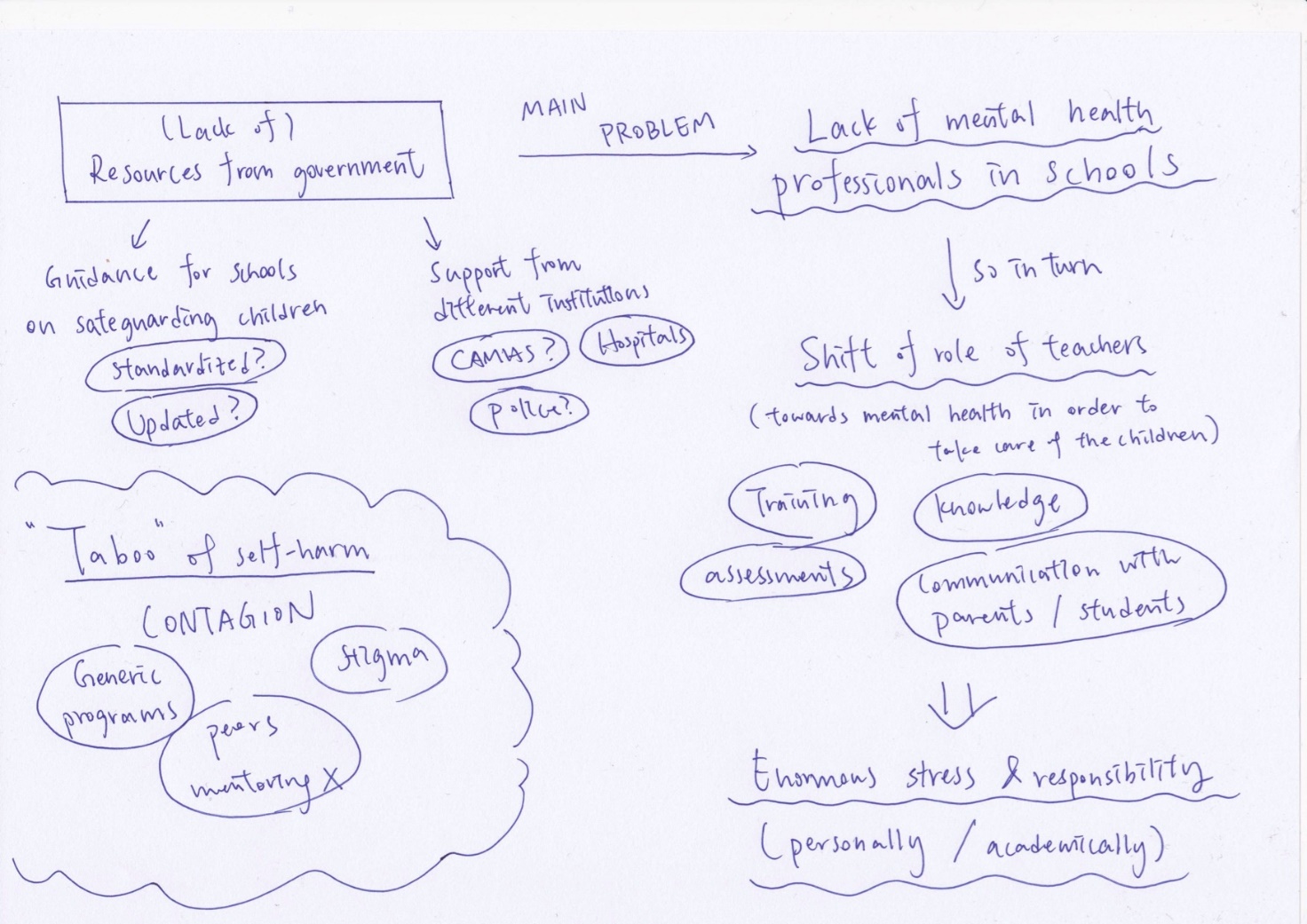


## **Supporting Information S3: Protocols or handbooks from different countries or local authorities for staff to respond safely to youth self-harm.**

| Protocol or handbook | Provider/Location | Link and summary (as written on website) |
| --- | --- | --- |
| Self-Harm: Guidelines and resources for schools to help support students who self-harm or are at risk of engaging in self-harming behaviour in West Sussex | West Sussex County Council (2021) | <https://www.westsussexscp.org.uk/wp-content/uploads/2020/12/WSCC-Self-harm-guidance-and-tool-kit-for-schools-v3.pdf>   - Guidelines and resources for schools to help support students who self-harm or are at risk of engaging in self-harming behaviour in West Sussex |
| Supporting the wellbeing of staff working with self-harm and suicidal content | Samaritans (2020) | <https://media.samaritans.org/documents/Supporting_the_wellbeing_of_staff_working_with_self-harm_and_suicide_content_FINAL.pdf>   - Information sheet that provides guidance of sites and platforms on supporting staff and volunteers who are exposed to self-harm and suicide related content online. |
| Responding to Issues of self-harm and thoughts of suicide in young people | Welsh Government (2019) | <https://gov.wales/sites/default/files/publications/2019-08/responding-to-issues-of-self-harm-and-thoughts-of-suicide-in-young-people-guidance.pdf>   - Quick flowchart guide for self-harm or suicidal thoughts or behaviours - Step-by-step guidance for managing self-harm or suicidal thoughts in young people - Self-care and supervision for staff members |
| Managing self-harm: Practical guidance and toolkit for schools in Cornwall and the Isles of Scilly | Turning the Tide (2017), powdered by NHS Kernow and Cornwall Council | <https://www.cornwall.gov.uk/media/jxqm25zm/managing-selfharm-guidance-and-toolkit-for-schools.pdf>   - Practical guidance and toolkit for schools - Assessing risk and other factors to consider when communicating with young people who self-harm |
| Young people who self-harm: A Guide for School Staff | University of Oxford , Charlie Waller Memorial Trust (2016) | <https://www.rcpsych.ac.uk/docs/default-source/improving-care/nccmh/suicide-prevention/wave-1-resources/young-people-who-self-harm-a-guide-for-school-staff.pdf?sfvrsn=e6ebf7ca_2>   - General information of self-harm and how staff could approach self-harm (helpful lead questions and statements) - Resources for students, parents and schools. |
| Working with young people with complex mental health issues | Project Air Strategy (2016) powered by New South Wales Department of Education and NSW Ministry of Health | <https://documents.uow.edu.au/content/groups/public/@web/@project-air/documents/doc/uow250430.pdf>   - Fact sheet on understanding and responding to emerging personality disorder trauma history, self-harm and suicidal behaviour and difficulties with identity, emotions and relationships |
| Self-harm: Guidance for staff within school and residential settings in Oxfordshire | Oxfordshire Adolescent Self Harm Forum (2016), powdered by Oxfordshire County Council, NHS Oxfordshire and Oxfordshire Safeguarding Children Board | <https://schools.westsussex.gov.uk/Page/18281>   - General information of self-harm and how staff could approach self-harm - Flowchart guide for self-harm or suicidal thoughts or behaviours |
| Non-Suicidal Self-Injury in Schools: Developing & Implementing School Protocol | Cornell Research Program on Self-Injury and Recovery (2010) | <http://www.selfinjury.bctr.cornell.edu/documents/schools.pdf>   - For schools staff and faculty, specifically for school administrators nurses - Includes information on developing and implementing a protocol, with a flowchart to aid decision making. |
| On Edge | NHS Greater Glasgow and Clyde | <https://www.seemescotland.org/young-people/resources/partner-resources/>   - Toolkit designed for adults who work with young people who self-harm - Four lesson plans to give pupils an overview of self-harm and support that is available |
| Young People and Self-Harm : Guidance for Schools | Nottinghamshire County Council and CAMHS | <https://nscp.nottinghamshire.gov.uk/media/bzxl52sd/young-people-and-self-harm-guidance-for-schools-10-17.pdf>   - Guidance for school staff working with young people around the issue of self-harm |

## **Supporting Information S4: Training programs from different charity sectors or non-governmental organisations on how to respond safely to youth self-harm.**

| Provider | Summary (as written on website) |
| --- | --- |
| NESSie | https://nessieined.com/training-and-supervision   - Training or supervision for parent and school on handling children with emotional or behavioural difficulties including self-harm |
| Developing Emotional Awareness and Listening (DEAL) by Samaritans | https://www.samaritans.org/how-we-can-help/schools/deal/   - Online toolkit and training sessions for educational professionals - Staff could support and build resilience in young people in the long term |
| Peer Education Project by the Mental Health Foundation | https://www.mentalhealth.org.uk/projects/peer-education-project-pep   - Evidence-based, universal mental health education programme that is available to all secondary schools in the UK and international British Schools - First train the staff members, then they could deliver lessons to pupils |
| Time to change Campaign | https://www.time-to-change.org.uk   - Useful resources including sessions, activities and training programs that aims to promote mental awareness among staff and students. - Note: this campaign has ended since March 2021 but resources are still present on the website |
